# *SpoTyping*: fast and accurate *in silico* Mycobacterium spoligotyping from sequence reads

**DOI:** 10.1186/s13073-016-0270-7

**Published:** 2016-02-17

**Authors:** Eryu Xia, Yik-Ying Teo, Rick Twee-Hee Ong

**Affiliations:** NUS Graduate School for Integrative Sciences and Engineering, National University of Singapore, Singapore, Singapore; Centre for Infectious Disease Epidemiology and Research, Saw Swee Hock School of Public Health, National University of Singapore, Singapore, Singapore; Department of Statistics and Applied Probability, National University of Singapore, Singapore, Singapore; Life Sciences Institute, National University of Singapore, Singapore, Singapore; Genome Institute of Singapore, Singapore, Singapore

## Abstract

*SpoTyping* is a fast and accurate program for *in silico* spoligotyping of *Mycobacterium tuberculosis* isolates from next-generation sequencing reads. This novel method achieves high accuracy for reads of both uniform and varying lengths, and is about 20 to 40 times faster than *SpolPred. SpoTyping* also integrates the function of producing a report summarizing associated epidemiological data from a global database of all isolates having the same spoligotype. *SpoTyping* is freely available at: https://github.com/xiaeryu/SpoTyping-v2.0.

**Electronic supplementary material:**

The online version of this article (doi:10.1186/s13073-016-0270-7) contains supplementary material, which is available to authorized users.

## Background

Tuberculosis (TB), caused mainly by *Mycobacterium tuberculosis* (*Mtb*), is a top infectious disease killer around the world and remains an acute international health problem, resulting in an estimated 9.6 million new cases and 1.5 million deaths globally in 2014 [1]. The global emergence and spread of drug-resistant TB have compounded the difficulty of treating and eradicating this disease.

Spoligotyping (spacer oligonucleotide typing) is a widely used genotyping method for *Mtb*, which exploits the genetic diversity in the clustered regularly interspersed short palindromic repeats (CRISPR) locus, which is also known as the direct repeat (DR) locus in *Mtb* genome [2]. Each DR region consists of several copies of the 36 bp DR sequence, which are interspersed with 34 bp to 41 bp non-repetitive spacers [3]. A set of 43 unique spacer sequences is used to classify *Mtb* strains based on their presence or absence. The patterns of presence and absence in each of the 43 spacer sequences can be summarized with a 43-digit binary code with 1 denoting the presence and 0 denoting the absence for each spacer, which can also be translated into a 15-digit numerical code [4] termed as the spoligotype. Spoligotypes can be used to compare *Mtb* isolates collected between different laboratories and countries. Spoligotyping is traditionally conducted using the PCR-based reverse line hybridization blotting technique [2]. Various new methods have recently been proposed for spoligotyping, the most of which are microarrays, such as the PixSysn QUAD 4500 Microarrayer [5], DNA microarray [6], hydrogel microarray (biochip) [7], *Spoligorifytyping* [8], and its follow-up TB-SPRINT [9]. Other spoligotyping methods include those based on a matrix-assisted laser desorption/ionization time-of-flight mass-spectrometry (MALDI-ToF MS) platform [10, 11]. Spoligotyping has also been applied to strain typing in other bacteria species such as *Legionella pneumophila* [12], *Campylobacter jejuni* [13, 14], and *Salmonella* [15].

Technological advancements in next-generation sequencing provide single nucleotide resolution for *Mtb* phylogenetic studies by allowing the construction of a single nucleotide polymorphism (SNP)-based phylogenetic tree. However, genotyping of bacteria is still needed for fast strain identification and correlation to previous isolates. For previous isolates, particularly the historical isolates, genotypes including spoligotypes may have been determined but whole genome sequences are not available and some isolates are not able to be sequenced. Under such circumstances, *in silico* genotyping from the whole genome sequences is necessary for correlating current isolates with previously genotyped ones. Several molecular genotyping techniques exist for *Mtb*, of which the most widely used are: (1) spoligotyping; (2) mycobacterial interspersed repetitive units - variable numbers of tandem repeat (MIRU-VNTR); and (3) IS*6110*-based restriction fragment length polymorphism (IS*6110*-RFLP) [16]. The inference of MIRU-VNTR from next-generation sequencing reads involves resolving the tandem repeats, which is extremely challenging for the current short sequence reads generated by the most widely used sequencing platforms. IS*6110*-RFLP commonly has its result based on the DNA fragment blots on electrophoresis gel image and thus focuses on the determination of the fragment lengths, which is also extremely challenging to infer since short read sequencing cannot be used alone to construct finished genomes. Spoligotyping, therefore, provides a unique opportunity to obtain the same result from whole genome sequences as the molecular genotyping result achieved in laboratories, which can correlate the isolates investigated using different approaches. *In silico* spoligotyping is also important for investigations using public data, where sequencing reads or complete genomic sequences are available but the spoligotypes of the isolates are not reported.

*SpolPred* [17] is a tool that accurately predicts the spoligotype of *Mtb* isolates from sequence reads of uniform length obtained from platforms such as Illumina GAII and HiSeq. However, for reads produced by platforms marketed for clinical diagnostics such as Illumina MiSeq and Ion sequencers, where the throughput is moderate and length of the reads are non-uniform, the accuracy of *SpolPred* is significantly reduced. *SpoTyping* improves the performance of *SpolPred* in three ways: (1) *SpolPred* reads in a fixed number of bases from each sequencing read as specified by the user. As a result, for sequencing experiments with non-uniform read length, the accuracy of prediction is highly dependent on the choices of the read length by the users. *SpoTyping*, by reading in the full length of the reads, makes use of all the available sequence data. (2) *SpolPred* requires the user to specify a direction for the reads, which can be either direct or reverse. However, since each FASTQ file consists of both direct and reverse reads, *SpolPred* only utilizes a fraction of the input sequence reads which can lead to incorrect predictions for sequencing experiments with low throughput. *SpoTyping* explicitly considers the reads in both directions, thereby using all the information presented in the sequence reads. (3) *SpolPred* relies on an inefficient sequence search algorithm, whereas *SpoTyping* integrates the BLAST algorithm in the search which reduces the time of the search considerably. In addition to the improvements listed above, *SpoTyping* also comes with novel functions not previously found in *SpolPred* or other software: (1) For TB disease outbreak investigation, it is necessary to quickly identify isolates with matching spoligotypes. *SpoTyping* thus automatically queries SITVIT [18], a global *Mtb* molecular markers database to retrieve associated epidemiological data for isolates with matched spoligotypes in an Excel spreadsheet, which can be presented as a graphical report showing the distribution summaries of the meta-data corresponding to the clades, years, and countries of isolation for these isolates. (2) *SpoTyping* works on different input files such as next-generations sequencing reads in FASTQ format, and complete genomic sequences or assembled contigs in FASTA format. (3) *SpoTyping* can be run on most operating systems such as Windows, Linux, and Mac OS, either as a non-interactive script which can be integrated into individual analysis pipelines or as an interactive application with a graphical user interface. Thus, we believe *SpoTyping* would be a useful tool for public health surveillance and genotyping from next-generation sequencing data in microbiological clinical diagnostic of *Mtb* strains.

## Implementation

**Fig. 1:**
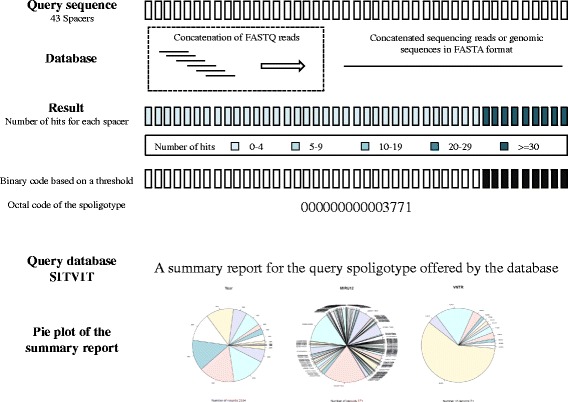
A schematic representation of the *SpoTyping* workflow. If the specified input contains sequencing reads, *SpoTyping* first concatenates the sequencing reads to form an artificial sequence. The artificial sequence, or genetic sequences when the input contains complete genomic sequence or assembled contigs, would be built into the BLAST database. After querying the 43 spacer sequences in the database, the results are parsed to count the number of hits for each spacer sequence. A hit threshold is set to define a spacer as ‘present’ in the genome, resulting in a 43-digit binary code with 1 as present and 0 as absent, which is further translated into the octal code of the spoligotype. The SITVIT database is then queried to identify matching isolates having the same spoligotype, where the associated data of the matched isolates are downloaded and summarized as pie charts

*SpoTyping* is implemented with Python and accepts two kinds of input files: single-end or pair-end sequence reads in FASTQ format, and complete genomic sequences or assembled contigs in FASTA format. A schematic representation of the *SpoTyping* workflow is shown in Fig. 1. When the input files are sequence reads, *SpoTyping* first concatenates all sequence reads in the input FASTQ file(s) into a single contiguous sequence in FASTA format which would be constructed into a BLAST [19] nucleotide database. The current program default (enabling the swift mode) is to read in no more than 250 Mbp of the sequence reads, which corresponds to a read depth of approximately 55X of the *Mtb* genome and would be sufficient in most situations. Disabling the swift mode would require *SpoTyping* to utilize all sequence reads with increased execution time. The set of 43 spacer sequences, each of 25 bp in length, would be queried against the constructed database using the standard nucleotide BLAST program. The BLAST output is then parsed to determine the number of hits for each spacer sequence in the input file(s). A maximum of one mismatch out of 25 bp of the spacer sequence is allowed for a BLAST match to be considered as a hit. For sequence reads, if a spacer sequence is absent in the *Mtb* isolate, then no or very few hits would be identified, while if the number of hits exceeds a threshold (hit threshold has a default of five error-free hits and six 1-error-tolerant hits), it indicates the presence of the spacer sequence where the number of hits correlates with the sequence read depth of the locus. For genomic sequences or assembled contigs, the presence of one hit for a spacer sequence indicates the presence of the spacer. The binary string of 43 digits, each digit representing one of the 43 spacer sequences with 0 indicating absence and 1 indicating presence, can therefore be written into an octal code that defines the spoligotype of the *Mtb* isolate. The predicted spoligotype is then automatically queried in the SITVIT database to retrieve all reported isolates having identical spoligotypes, where associated data corresponding to the MIRU12, VNTR, SIT, MIT, VIT, clades, countries of origin, countries of isolation, and year of report for these isolates would be downloaded in an Excel spreadsheet. *SpoTyping* also includes an R script that can present summary statistics of the associated meta-data as a pdf report.

The accuracy of *SpoTyping* was assessed in comparison with *SpolPred* on three datasets: (1) 161 isolates sequenced on Illumina HiSeq (SRA: SRA065095); (2) 30 isolates sequenced on Illumina MiSeq (ENA: PRJNA218508); and (3) 16 isolates sequenced on Ion Torrent (ENA: PRJEB6576). The first assessment was conducted on a dataset of 161 *Mtb* isolates sequenced on Illumina HiSeq with experimentally determined spoligotypes reported [20]. Both *SpoTyping* and *SpolPred* were run with default parameters. The predicted octal codes were each queried in the SITVIT database to identify the matching spoligotype for comparison with the reported spoligotype. Discordant results were examined by searching the spacer sequences on the contigs assembled using the *de novo* assembly software *Velvet* [21]. The next assessment was conducted on a dataset of 30 *Mtb* isolates sequenced on Illumina MiSeq without reported spoligotypes. The reference spoligotype for each isolate was determined by manual inspection of the BLAST output file to determine the number of hits for each spacer sequence in the sequence reads. Given that the sequence read depths are above 20X for all isolates, no hit for a spacer sequence is a strong indication of its absence while a number of above five hits is a strong indication of the presence of the spacer sequence. While a judgement cannot be safely made based on a hit number of 1 to 5, isolates with at least one such case were removed from the study, leaving only isolates with confident reference spoligotypes. *SpoTyping* was run with default parameters while *SpolPred* calls for a specified read length, where a range of read lengths were used based on the read length percentiles from 0.04 to 1 at a step of 0.04, resulting in a total of 25 predictions for each isolate. The accuracy of *SpoTyping* was also assessed in comparison with *SpolPred* on a dataset of 16 *Mtb* isolates sequenced on Ion Torrent. The reference spoligotypes were determined similarly as those for Illumina MiSeq data. The running parameters were also similar as those for Illumina MiSeq data.

The time performance of *SpoTyping* was compared with *SpolPred* based on the first dataset. The programs were run on a 64-bit Fedora Linux server workstation having a 2.0 GHz quad processor and 32 GB RAM. Both *SpoTyping* and *SpolPred* were run twice for each isolate either with or without the swift mode. Default parameters were used for *SpoTyping* swift mode, while for non-swift mode, 10 error-free hits or 12 1-error-tolerant hits (options of -m 10 -r 12) was taken as the hit threshold due to the high sequencing coverage to eliminate false positives. For *SpolPred*, the pair-end sequence reads were first concatenated (concatenation time not counted toward the running time). The read lengths were set to be the actual read lengths. The hit threshold was similarly set to be 10 (option of -m 10) in the non-swift mode.

The performance of *SpoTyping* was assessed for various sequence read depths to determine its applicable range, where we determined the accuracies of the *SpoTyping* prediction for: (1) an H37Ra *Mtb* isolate which had a sequencing throughput of 3,000 Mbp (approximately 670X); and (2) a Beijing-genotype *Mtb* isolate with a sequencing throughput of 2,700 Mbp (approximately 600X) by performing 50 iterations each for six down-sampling ratios of 50 %, 20 %, 10 %, 5 %, 2 %, and 1 % of the initial number of reads for each isolate. In each down-sampling experiment, a certain percent of the sequence reads were randomly selected from the original FASTQ file to form a new file with a lower read depth, where the percentage is called the down-sampling ratio. For all of the down-sampling experiments, default settings were used except for the categories of 2 % and 1 % where the hit threshold was set to two error-free hits and three 1-error-tolerant hits (options of -m 2 -r 3). The false positives caused by the concatenation of sequence reads were also assessed in the down-sampling experiment.

The selection of the hit thresholds was also based on the down-sampling experiments. In each down-sampling experiment, the number of both error-free hits and 1-error-tolerant hits for each spacer identified by *SpoTyping* were divided by the estimated read depth (number of sequence bases/ 4,500,000) of the experiment, representing the number of hits as a percentage of the estimated read depth. For each spacer in each experiment, the percentage is used as the feature to classify a spacer as present or absent, while the spacer’s actual class of presence or absence is used to assess whether the classification is correct. A set of percentages was used as the thresholds to calculate the respective true positive rates and false positive rates, which were plotted as a receiver operating characteristic (ROC) curve. The thresholds were selected to maximize the true positive rate while minimizing the false positive rate.

The Beijing-genotype isolate can be accessed through the European Nucleotide Archive (ENA) code ERP006354. The H37Ra isolate is a laboratory strain that was sequenced as part of a validation sequencing run, and the FASTQ files will be provided upon request.

## Results

### *In silico* spoligotyping of 161 *Mtb* isolates sequenced on Illumina HiSeq

For all the 161 *Mtb* isolates, *SpoTyping* and *SpolPred* predicted the same spoligotypes (Additional file 1: Table S1), of which 20 isolates either without a match in the SITVIT database or reported as ‘New’ were excluded from subsequent comparisons. Of the remaining 141 isolates, predictions of *SpoTyping* and the laboratory determined spoligotypes for 127 isolates (90.07 %) were identical. For the 14 discordant isolates, the spacer sequences were searched in the assembled contigs to determine the spoligotypes, which are all concordant with the predictions from *SpoTyping* (Additional file 1: Table S2).

### *In silico* spoligotyping of 30 *Mtb* isolates sequenced on Illumina MiSeq

**Fig. 2:**
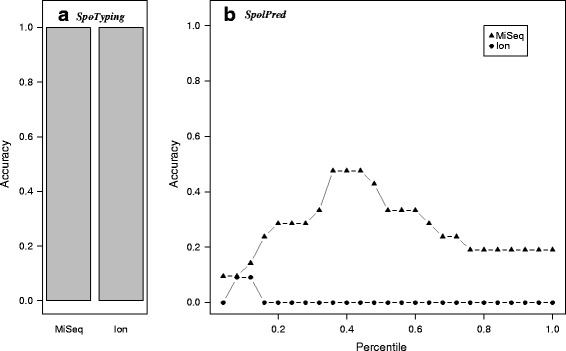
Prediction accuracy of *Mtb* isolates sequenced on Illumina MiSeq and Ion Torrent. *SpolPred* requires a read length to be specified which results in inconsistent predictions for different specifications. The accuracy assessment was conducted between *SpoTyping* (**a**) and *SpolPred* (**b**) on 21 MiSeq-sequenced isolates and 11 Ion-sequenced isolates, with *SpoTyping* predictions using default parameters and *SpolPred* predictions using different read length percentiles as the input read lengths. While *SpoTyping* have perfect accuracies for both datasets, *SpolPred* gives varying accuracies depending on the read length, which are always lower than 50 %

The accuracy of *SpoTyping* was then assessed in comparison with *SpolPred* on 30 *Mtb* isolates sequenced on Illumina MiSeq, among which 21 passed filtering for having reference spoligotypes confidently determined. *SpoTyping* correctly inferred the spoligotypes for all 21 isolates. Since *SpolPred* requires for a read length to be specified as input, a range of read lengths were assessed based on the percentiles from 0.04 to 1 at a step of 0.04, resulting in a total of 25 predictions for each isolate whose read length specifications are summarized in Additional file 1: Table S3. At each percentile, the predictions for the 21 isolates were analyzed to calculate the prediction accuracy, which is summarized in Fig. 2 and Additional file 1: Table S4. *SpolPred* performs the best using the read lengths at the 0.36, 0.40, or 0.44 percentiles, with accuracies around 50 %. The prediction accuracy of *SpolPred* is significantly lower than that obtained by *SpoTyping* and is also highly dependent on the choice of read length used as input which in itself is difficult to determine.

### *In silico* spoligotyping of 16 *Mtb* isolates sequenced on Ion Torrent

The accuracy for spoligotype inference was also determined on 16 *Mtb* isolates sequenced on Ion Torrent with spoligotypes reported to be all Beijing genotype [22]. Of the 16 isolates, 11 have confidently determined spoligotypes, which are all of the spoligotype ‘000000000003771’ as are consistent with the reported Beijing genotype. *SpoTyping* makes correct prediction for all the 11 isolates. The performance of *SpolPred* is summarized in Fig. 2, Additional file 1: Table S5 and Table S6. *SpolPred* performs best using the read length at the 0.08 and 0.12 percentile, with accuracies of only around 10 %.

### Comparison of time performance for *SpoTyping* and *SpolPred* on 161 *Mtb* isolates

For the 161 *Mtb* isolates tested, *SpoTyping* is about 20 to 40 times faster than *SpolPred*, with *SpoTyping* taking an average of 28.8 s (standard deviation is 5.3 s) in its swift mode, and an average of 56.4 s (standard deviation is 8.0 s) to process all reads, while *SpolPred* took an average of 17 min 19.3 s (standard deviation is 1 min 35.3 s) by using the -s option, or an average of 18 min 20.0 s (standard deviation is 50.2 s) to process all reads (Additional file 1: Table S1).

### Down-sampling experiments

**Fig. 3:**
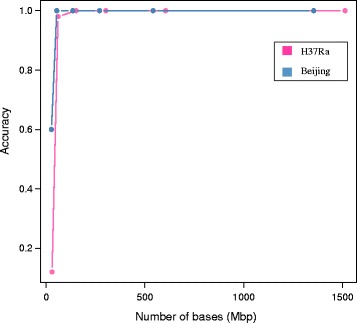
Assessing the accuracy of *SpoTyping* across various sequence read depths for H37Ra and Beijing-genotype isolates. With blue points denoting the Beijing genotype, pink points denoting H37Ra, the prediction accuracies were assessed with the sequencing throughput measured by the number of bases for all the down-sampling experiments. *SpoTyping* is suitable for sequencing runs with throughput over 54 Mbp (estimated depth of approximately 12X), where the accuracy is almost 100 %

Based on the down-sampling experiments which first explore the applicable throughput for accurate spoligotype inference, *SpoTyping* is able to efficiently and accurately predict the spoligotype for isolates having sequencing throughput over 54 Mbp (read depth of approximately 12X) with accuracies above 98 % (Fig. 3, Additional file 1: Table S7 for H37Ra and Additional file 1: Table S8 for Beijing). However, for isolates that are sequenced at very low coverage (below 10X), using the lower threshold is still not sufficient to make accurate predictions as some of the spacer sequences would not be adequately sequenced and represented in the input FASTQ file(s).

Since *SpoTyping* concatenates sequence reads into an artificial sequence to create the BLAST database, an immediate concern is the false positives created due to chimera sequences. In all of 600 down-sampling experiments performed for both H37Ra and Beijing genotype *Mtb* isolates, the maximum number of false positive hit is 1 for both error-free hits and 1-error-tolerant hits. Of the experiments, 98.3 % (590/600) show no false positive error-free hits while 95.7 % (574/600) show no false positive 1-error-tolerant hits. The likelihood of false positives created due to chimera sequences is thus low which can be further reduced by setting more stringent hit thresholds.

### Threshold selection

**Fig. 4:**
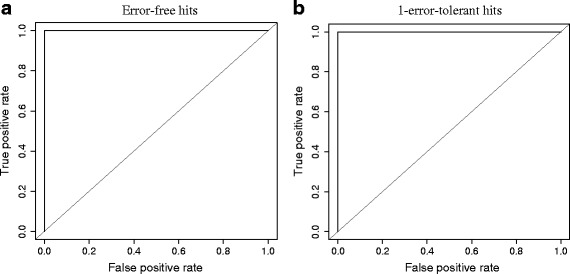
ROC curves for the selection of hit thresholds. The ROC curves were plotted for both error-free hits (**a**) and 1-error-tolerant hits (**b**) to select the hit thresholds. Diagonal lines, also known as lines of no discrimination, were plotted as references of random guess. The threshold evaluation was based on a percentage calculated as the number of hits divided by the estimated read depth. A set of percentages was used as the thresholds to calculate the respective true positive rates and false positive rates, which were plotted as the ROC curves. Both ROC curves show constantly high true positive rates and low false positive rates, with the areas under the ROC being 0.9999997 and 0.9999998, respectively

We evaluated the choice of the hit thresholds to determine the presence or absence of a spacer sequence used in *SpoTyping*. The evaluation was conducted in the down-sampling experiments, based on the groups with down-sampling ratios from 2 % to 50 % (read depths between approximately 12X and approximately 300X) where accurate inferences for the spacer sequences are possible to be made. A total of 21,586 spacer sequence instances ((5 down-sampling ratios * 50 rounds for each down-sampling ratio * 43 spacer for each round + 43 spacers without down-sampling) = 10,793 spacers for each of the two strains) with their respective number of hits identified by *SpoTyping* were included in the analysis, of which 10,040 are absent cases and 11,546 are present cases. The number of hits was divided by the estimated read depth to represent the number of hits as a percentage of the read depth in order to adjust for the difference in sequencing throughput. A set of percentages was used as the thresholds to calculate the respective true positive rates and false positive rates, which were plotted as an ROC curve (Fig. 4). The ROC curves for both the error-free hits (Fig. 4a) and 1-error-tolerant hits (Fig. 4b) show very high true positive rates and very low false positive rates, with the areas under the ROC being 0.9999997 and 0.9999998, respectively. False positive rates are always nearly 0, while the true positive rates are above 99 % by setting the thresholds to be 1.80 % to 14.86 % of the read depth for error-free hits and 1.80 % to 14.88 % of the read depth for 1-error-tolerant hits. Thus the default thresholds of five error-free hits and six 1-error-tolerant hits are applicable to sequencing experiments with estimated read depths between approximately 30X and approximately 280X. The thresholds can be adjusted accordingly given sequencing throughputs beyond this range.

## Discussion

The increasing global burden of TB, especially drug-resistant strains, has put a significant spotlight on pathogen whole genome sequencing as a rapid diagnostic tool, which is of great relevance to both public health surveillance and clinical treatment. The application of next-generation sequencing in clinical microbiology requires fast and easy-to-use software that is able to accurately produce easily comprehensible results. As shown, *SpoTyping* is able to accurately determine the spoligotype of the *Mtb* isolate rapidly. Contrary to *SpolPred* which is sensitive to the user-specified read length and gives inconsistent predictions at different read lengths, *SpoTyping* gives accurate predictions based on sequence reads produced from different technologies regardless of the length uniformity of the sequence reads and is 20 to 40 times faster than *SpolPred*. The additional functions of database query, information visualization and report generation provided by *SpoTyping* where the predicted spoligotype is automatically queried in the SITVIT database to retrieve all associated epidemiological data corresponding to the MIRU12, VNTR, SIT, MIT, VIT, clades, countries of origin, countries of isolation, and year of report and presented as a report would be a useful tool for public health surveillance of *Mtb* strains causing tuberculosis.

While there are several molecular typing techniques for *Mtb*, the most widely used are spoligotyping, MIRU-VNTR, and IS6110-RFLP. Spoligotyping, though being a relatively simple, cost-effective, and high-throughput method, suffers from the limitations of: (1) having relatively low discriminatory power [23] due to its use of only a single genetic locus; and (2) having limited use in phylogenetic study. Among the genotyping methods for *Mtb*, a combination of spoligotyping and MIRU-VNTR was reported to be the best strategy [24, 25]. However, significant technical challenges currently exist for the accurate *in silico* typing from next-generation sequencing reads of MIRU-VNTR which involves resolving tandem repeats and IS6110-RFLP whose result is based on DNA fragment blots on electrophoresis gel image and thus involves the determination of the fragment lengths. Spoligotyping, as a result, provides a unique chance to obtain the same result from whole genome sequences as the molecular typing result achieved in laboratories, which can correlate the isolates investigated with different approaches. Though spoligotyping has less discrimination power than SNP phylogeny inferred from whole genome sequences, it is unique in correlating the genomic data produced in research labs and the molecular typing data from clinical laboratories. Thus *in silico* spoligotyping is not only a genotyping method for *Mtb* isolate differentiation, but also a bridge between isolates with whole genome sequences available and isolates typed and investigated with traditional laboratory protocols, especially those historical isolates that are not sequenced. Inexorably, clinical surveillance and management of TB, particularly for disease diagnosis and treatment, will progress towards the use of direct *Mtb* sequencing. Thus the ease of use and interpretability of the results will be of considerable importance to users within a clinical setting, which is well achieved with *SpoTyping*.

A recently published letter reported CASTB, an analysis server for the *Mycobacterium tuberculosis* complex, which provides next-generation sequencing data analysis tools for virtual typing (spoligotyping included), virtual drug resistance analysis, and phylogenetic analysis [26]. While the webserver provides a comprehensive overview at the sequencing data, the performance of each tool is not well evaluated in the publication. More accurate and well assessed tools are thus needed for further analysis. *SpoTyping* is well assessed to provide high accuracy for *in silico* spoligotyping and thus demonstrates the reliability of the results. *SpoTyping* also benefits from its open source nature that it can be easily integrated into in-house analysis pipelines for in-depth analysis of the sequencing data. When talking about execution time, services provided by web servers may be very slow due to the inherent issues such as the process of data uploading and the availability of the computational resources. *SpoTyping*, on the other hand, can be set up locally and provides the spoligotyping result within a minute.

For the 14 discordant spoligotypes between the laboratory tests and the *in silico* predictions made by *SpoTyping* in the 161 *Mtb* isolates sequenced on Illumina HiSeq, the SNP-based phylogenetic tree of these 161 Mtb isolates in the original article [20] was examined to compare the lineage with the spoligotyping results (Additional file 1: Table S9). Out of the 14 discordant results, three showed better concordance of the *in silico* prediction with the lineage on the tree. As an example, an isolate (Accession: SRR671868, Strain: 143) located at Lineage 4.2 on the SNP-based phylogenetic tree is reported to be a Beijing genotype based on the laboratory test in the publication, while predicted to be a T2 genotype by *SpoTyping*. However, the Beijing genotype is usually found at East Asia Lineage 2, while Lineage 4 usually harbors the Euro-American genotypes. One of the discrepancies may be caused by the different naming of spoligotypes in different databases (Beijing and Beijing-like). Definite conclusion cannot be made for the remaining 10 isolates for which the reported spoligotype and *in silico* predicted spoligotype are different while the lineages for both spoligotypes are similar (T2 and H3, for example). For such isolates, the difference could be due to the discrepancy between laboratory tests and the genomic features.

## Conclusions

*SpoTyping* is an accurate, fast, and easy-to-use program for *in silico* spoligotyping of *Mtb* isolates from next-generation sequencing reads, complete genomic sequences, and assembled contigs. In addition, *SpoTyping* automatically queries the global *Mtb* molecular markers database SITVIT to retrieve associated data for matching isolates with the inferred spoligotypes, which can be summarized graphically to generate a report. *SpoTyping* would be a useful tool for public health surveillance and genotyping of *Mtb* strains.

## Availability and requirements

- **Project name:** SpoTyping
- **Project home page:**https://github.com/xiaeryu/SpoTyping-v2.0
- **Operating systems:** Linux, Mac OS, Windows
- **Programming language:** Python (version 2.7)
- **Other requirements:** BLAST
- **License:** GNU General Public License
- **Any restrictions to use by non-academics:** None

## Additional file

Additional file 1: Table S1. Spoligotype prediction and time performance of 161 *Mtb* isolates with *SpoTyping* in comparison with *SpolPred*. **Table S2.** Spoligotypes of 14 *Mtb* isolates determined from contigs obtained by *de novo* assembly, *Velvet*. **Table S3.** Actual length at different percentiles used as the read length for *SpolPred* spoligotype prediction of isolates sequenced on Illumina MiSeq. **Table S4.** Spoligotype prediction for *Mtb* isolates sequenced by Illumina MiSeq with *SpoTyping* in comparison with *SpolPred*. **Table S5.** Actual length at different percentiles used as the read length for *SpolPred* spoligotype prediction of isolates sequenced on Ion Torrent. **Table S6.** Spoligotype prediction for *Mtb* isolates sequenced by Ion Torrent with *SpoTyping* in comparison with *SpolPred*. **Table S7.** Statistics of time and accuracy of running *SpoTyping* on 50 iterations each for various down-sampling ratios of an H37Ra *Mtb* isolate. **Table S8.** Statistics of time and accuracy of running *SpoTyping* on 50 iterations each for various down-sampling ratios of a Beijing *Mtb* isolate. **Table S9.** Lineages of 14 discordant *Mtb* isolates by *SpoTyping*, experimentally reported and phylogenetic tree. (XLSX 55 kb)

## Abbreviations

CRISPR: clustered regularly interspaced short palindromic repeats
DR: direct repeat
MIRU-VNTR: mycobacterial interspersed repetitive units - variable numbers of tandem repeat
*Mtb*: *Mycobacterium tuberculosis*
RFLP: restriction fragment length polymorphism
ROC: receiver operating characteristic
SNP: single nucleotide polymorphism
TB: tuberculosis
